# Airway Management of Two Patients with Penetrating Neck Trauma

**Published:** 2009-06

**Authors:** P Bhattacharya, M C Mandal, S Das, S Mukhopadhyay, S R Basu

**Affiliations:** 1 PG Student, Department Of Anaesthesiology. North Bengal Medical College, P.O.-Susrutanagar, PIN-734012, District-Darjeeling, West Bengal, India; 2,3,4 Assistant professor, Department Of Anaesthesiology. North Bengal Medical College, P.O.-Susrutanagar, PIN-734012, District-Darjeeling, West Bengal, India; 5 Professor and Head, Department Of Anaesthesiology. North Bengal Medical College, P.O.-Susrutanagar, PIN-734012, District-Darjeeling, West Bengal, India

**Keywords:** Penetrating neck trauma, Open tracheal injury, Airway emergency, Airway management

## Abstract

**Summary:**

Direct trauma to the airway is a rare injury which can lead to disastrous consequences due to compounding effect of bleeding, aspiration of blood, airway obstruction and severe sympathetic stimulation. Here we are presenting two cases of open tracheal injury in two adult males following assault with sharp weapon. Two different techniques of securing the airways were employed depending upon the severity and urgency of the situation. In the first case, orotracheal intubation helped the surgeon to repair airway around the endotracheal tube whereas in the second patient this stenting effect was absent as he was intubated through the distal cut-end of trachea in the face of airway emergency.

## Introduction

Penetrating neck trauma is responsible for 5% to 10% of all trauma admissions. These injuries are challenging and encountered as a small component of an anaesthesiologist's overall clinical experience. The neck contains a dense concentration of vital structures that are not always easy to assess by physical examination or surgical exploration.^1^ Irrespective of these problems the overall mortality is relatively low, ranging from 0 to 11%.^2^ Laryngeal injuries are quite rare due to the protection offered to the laryngeal apparatus by the mandible and cervical spine. However acute blunt laryngeal trauma can be a life threatening event and often poses a difficult airway problem.^3^

## Case report

We got two male patients in close proximity of time in our emergency department sustaining wide open wound in front of the neck at the level of cricothyroid membrane. They presented with frequent cough, dyspnoea, aphonia, haemoptysis, odynophagia, some degree of local surgical emphysema, occasional bouts of vomiting mixed with swallowed blood and were conscious. The first patient had pulse 114/min, BP 150/90 mmHg, clear chest, warm extremities. Expiratory air was coming out of the open tracheal wound, but there was neither visible active bleeding nor any inspiratory sucking. Whereas the second patient had worse haemodynamics (pulse 146/min, BP 90/60 mmHg), cold clammy extremities, coarse pulmonary crepitations and severe respiratory distress even in sitting position. The patient was breathing through the distal tracheal end which was retracted and could be identified by gush of expiratory air coming out of it. There was sucking-in of the blood clots during inspiration.

In emergency ward, wide-bore intravenous(iv) cannula was inserted, blood sample was sent for grouping and cross matching. In the operation theatre, ECG, NIBP and pulse-oximeter were attached. Patients were premedicated with iv ranitidine 50 mg, ondansetron 4 mg, glycopyrrolate 0.2 mg and fentanyl 50 mcg. Informed consent was taken.

**Fig-1:**
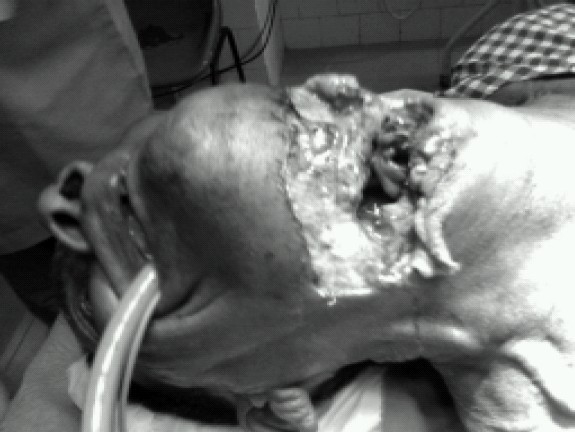
Showing the first patient intubated orotracheally.

**Fig-2:**
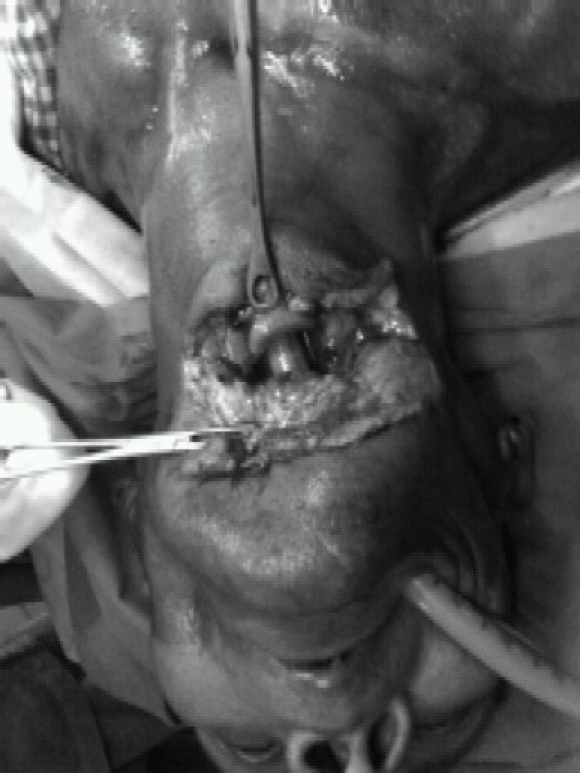
Showing the first patient intubated orotracheally. The tube came out of the proximal airway defect which was guided into the open distal tracheal rent.

We intubated the first patient orotracheally under topical anaesthesia with ten percent lidocaine spray. The tube came out of the proximal airway defect which was guided into the open distal tracheal rent (Fig-1 & 2). Anaesthesia was provided with fentanyl 2mcg/kg, propofol; vecuronium and O_2_/N_2_O. The surgical procedure involved primary repairs of the pharynx (around a nasogastric tube) and the airway defect [around the endotracheal tube (ETT)], followed by mid-tracheostomy.

**Fig-3:**
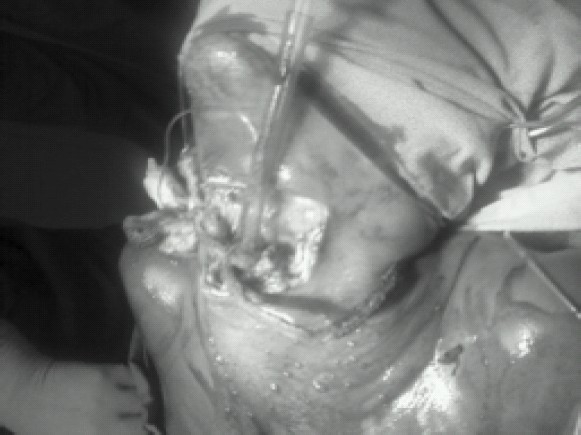
In the second patient, due to airway emergency, intubation was done through the widely gaped open wound.

**Fig-4:**
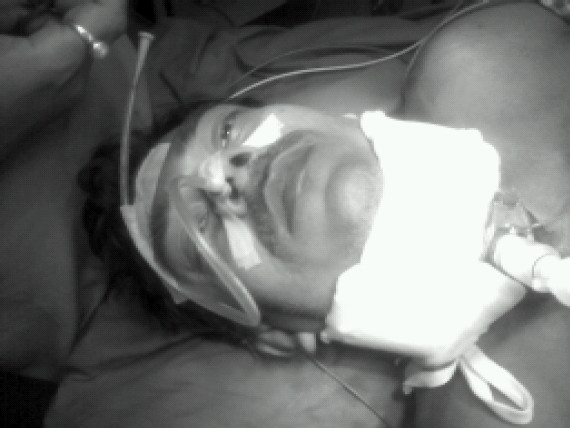
In the second patient, intubation through the open tracheal wound was followed by tracheostomy after which the endotracheal tube was removed and anaesthesia was aintained through tracheostomy wound.

In the second patient, due to airway emergency, we introduced a lubricated bougie through distal tracheal rent after topical anaesthesia. The tracheal rent was retrieved with Allis' forceps. We slid an endotracheal tube over the bougie. Intubation was followed by low-tracheostomy. (Fig 3, Fig-4). Then the endotracheal tube was withdrawn. Anaesthesia was provided with iv ketamine, vecuronium, fentanyl 2mcg/kg and O_2_/N_2_O. The tracheal end was sutured to the cricoid cartilage and repair of the oesophageal injury was done.

At the end of the procedure, both the patients were reversed satisfactorily. The patients were shifted to the recovery room with supplemental O_2_ through a T-piece via tracheostomy. Unwanted neck extension was prevented with a supporting cast behind the neck. Nutrition through nasogastric tube was maintained for 2 weeks. After 2 weeks, oesophagoscopy and fibreoptic laryngoscopy was done which confirmed fair recovery of soft tissues. Thereafter oral feeds were allowed. Tracheal decannulation was done after 3 weeks and the patient were discharged after 4 weeks. Both patients are still under monthly follow up and doing well, though the first patient having minor degree of dysphonia and the second patient is suffering from some problems with swallowing of solid foods.

## Discussion

Direct trauma to the airway is a rare injury, accounting for less than 1% of traumatic injury^4^. These injuries are quite challenging to the anaesthesiologist as these are compounded by great vessel injury, impending airway obstruction and severe sympathetic stimulation^5^. Most of the penetrating neck injuries (50-80 %) involve zone II of the neck i.e. from the cricoid cartilage to the angle of the mandible^4^,^5^. The prevailing site of tracheal transection is extrathoracic and involves the cricotracheal junction, because the connective tissues in this area are weak^6^. Injury at or below the cricoid carries the risk of partial or total airway obstruction with resultant asphyxiation and definite airway management of such patients is a life saving measure^4^. These patients can be managed with different airway techniques such as intubation through the visible airway defect, conventional orotracheal intubation, or tracheostomy.

The description and treatment of penetrating tracheo-oesophageal injuries was first reported in Scotland in 1792^7^. The open tracheal injuries most commonly result from violent crime, primarily of ballistic and knife injuries^4^. Penetrating injuries occur most commonly (75%) in the cervical trachea because of its exposed position.^6^,^8^ Despite severe tracheo-pharyngeal injuries, carotid sheath with its contents was spared in our two patients probably due to posteriorly tilted head pulling the carotid sheath behind a taut sternocleidomastoid which was injured instead. Injury to minor peripheral vessels may lead to significant hypotension, as occurred in our second patient and ligation of the bleeding vessels effectively prevented further degradation.

Hoarseness, subcutaneous emphysema, dyspnoea, dysphagia and haemoptysis strongly suggest disruption of laryngo-tracheal continuity^4^. Oesophageal injury frequently accompanies tracheal injury, as it is intimately associated with the trachea at all levels and may lead to fatal mediastinitis^6^,^9^. Controversy exists about immediate surgical exploration in asymptomatic patients, taking into account the significant number of negative explorations but there is consensus opinion regarding immediate exploration in cases of associated oesophageal injury, progressive subcutaneous or mediastinal emphysema, pneumothorax and severe dyspnoea requiring intubation.^5^ In any patient with neck injury, the first priority is to establish an airway^10^. If the patient is asphyxiating, the quickest way to secure the airway in a patient with open cervical wounds is to in-tubate the distal open end of the trachea through the wound itself followed by a tracheostomy and repair of the tracheal defect^4^,^6^. Occasionally, because of complete transection, the distal cut-end of the trachea may retract into thorax, which can be retrieved and intubated, as in our second patient^6^. Now, in the first patient, the airway was not severely compromised, but airway control was necessary nevertheless. Davari HR and Malekhossini SA mentioned that orotracheal intubation should be attempted in all such cases except those with massive maxillofacial trauma.^8^ Orotracheal intubation as a stent also helps in primary repair of the trachea. Sedative, hypnotic and opioid should be avoided or given cautiously and muscle relaxant should be employed only if there is capability to perform an immediate tracheostomy should intubation fail^4^,^11^. Oxygenation of these patients using face mask is not effective due to airway disruption^12^. Agitation, straining and coughing may result in increased intratracheal pressure, spread of subcutaneous emphysema, complete airway obstruction and increased venous pressure leading to dislodgement of clots and torrential bleeding^10^. Topical application of anaesthetic seems a suitable option, as field blocks can be difficult to perform in these cases.

Fibreoptic bronchoscopy allows identification of tracheal lumen and injury and helps in smoother entry of ETT through the cut-end of trachea but may not be feasible in emergency situation. Also blood, secretions and oedema can interfere with visualization^4^. Intubation by direct laryngoscopy would be ideal considering the speed with which it could be performed but surprises could always await and emergency surgical access should always be considered as another alternative^13^. The options for surgical management of injuries to extrathoracic trachea are primary repair, resection and anastomosis and tracheostomy and maintenance of neck flexion in the early postoperative period. Insertion of a tracheal graft may be necessary.

Postoperative mechanical ventilation may be required in many cases, but may disrupt the repaired trachea. Pain control is essential in order to provide adequate pulmonary ventilation, clearance of secretions and alleviation of sympathetic over-activity. Adequate systemic hydration, prophylactic nebulisation and humidification help to prevent drying up of secretions.

A new air leak, haemoptysis, worsening mediastinal or subcutaneous emphysema warrant bronchoscopy to assess suture line.
